# Synovial chondromatosis originating from the synovium of the anterior cruciate ligament: a case report

**DOI:** 10.1186/1758-2555-1-6

**Published:** 2009-04-23

**Authors:** Tokifumi Majima, Tamotsu Kamishima, Kouichi Susuda

**Affiliations:** 1 Department of Joint Replacement and Tissue Engineering, Hokkaido University Graduate School of Medicine, Sapporo, Japan; 2 Department of Radiology, Hokkaido University Graduate School of Medicine, Sapporo, Japan; 3 Shin-Sapporo Orthopedic Hospital, Sapporo, Japan

## Abstract

A case of synovial chondromatosis originating from the synovium of the anterior cruciate ligament (ACL) resulting in a mechanical block to knee extension is reported. A 36-year-old man complained of a restricted range of left-knee motion and pain when walking. Plain roentgenograms showed normal appearance, however, magnetic resonance imaging showed intensity changes in the ACL. Arthroscopically, numerous small free bodies were observed. Proliferation of synovium and cartilaginous tissues were identified around the ACL. There were no significant findings in the synovium except around the ACL. The synovium around the ACL was resected and free bodies were washed out. This is the first report of synovial chondromatosis originating from the synovium of the ACL.

## Background

Synovial chondromatosis is a relatively rare disease and large joints such as knee-joints, hip-joints, and elbow-joints are more commonly affected [1,2]. Among knee-joints, cases originating from the cruciate ligament of the knee are very rare and only 2 cases have been reported on presentation from the posterior cruciate ligament [3,4]. We experienced a case of synovial chondromatosis originating from the synovium of the anterior cruciate ligament (ACL) of the knee and report the results.

## Case report

A healthy 36-year-old male with no existing history or family history complained of pain in the left knee when in motion since 2000. Following diagnosis of a damaged meniscus in 2003, he received an intra-articular injection of hyaluronic acid at another institution. The symptoms were improved temporarily, but pain in the left knee reoccurred in the winter of the same year and locking-like symptoms were also expressed. We initially examined this patient with major complaints of pain in the left knee and a restricted range of left-knee motion in 2004. At the time of the initial visit, squatting was difficult and pain in the left-knee appeared when walking and climbing up or down a staircase. Swelling was detected in the left-knee joint and there was a restriction with an extension of -20° and a flexion of 120° in the range of left-knee motion (hereinafter referred to as ROM). Forceful extension of the knee was elastically inhibited and pain was felt at this time, presenting similar symptoms to locking at the time of meniscus damage. The results of Lachman test, anterior drawer test, and varus/valgus stress test were all negative as was the result of McMurray's test.

**Figure 1:**
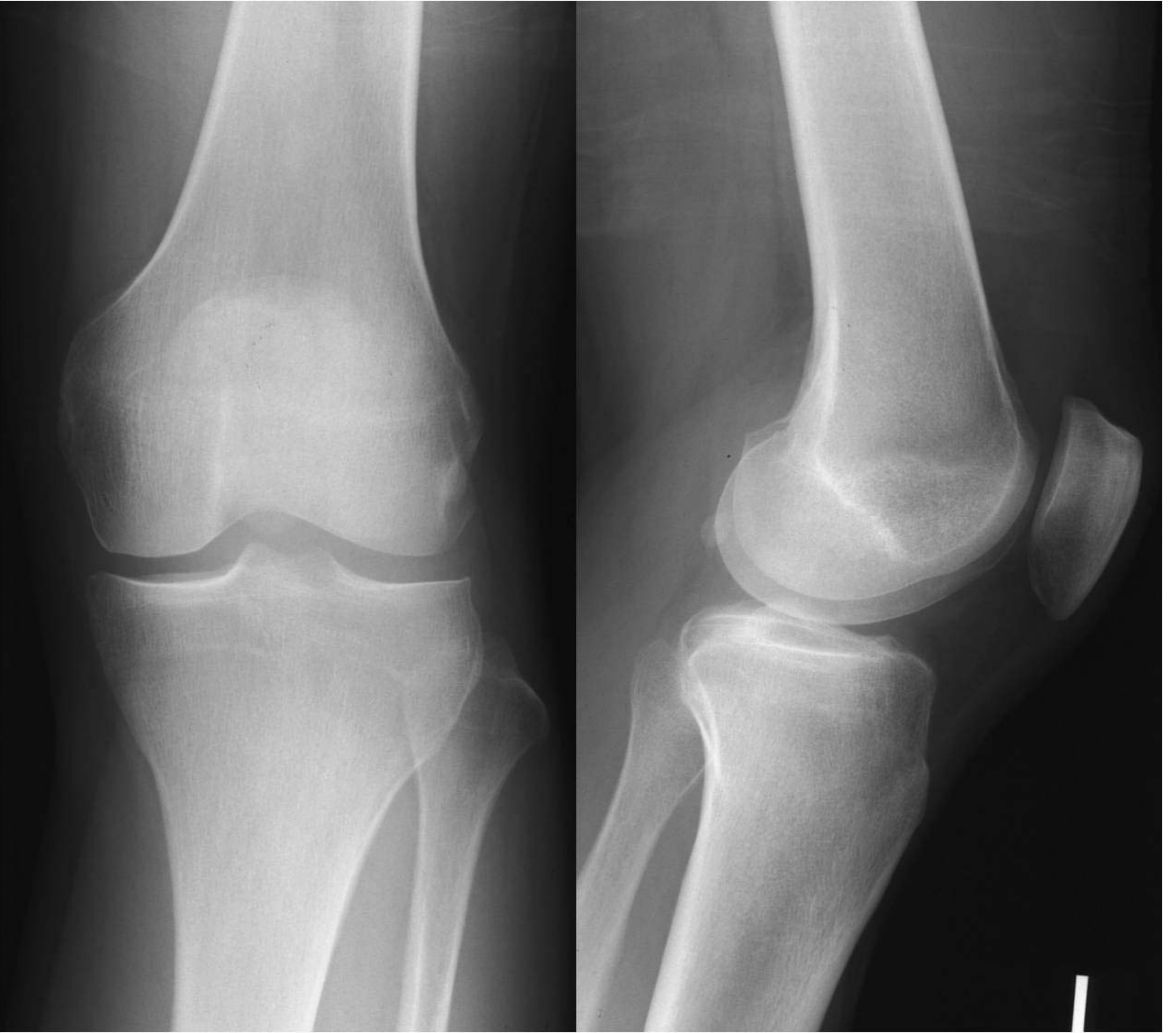
**Plain radiograph of the left knee at the time of initialvisit**.

**Figure 2:**
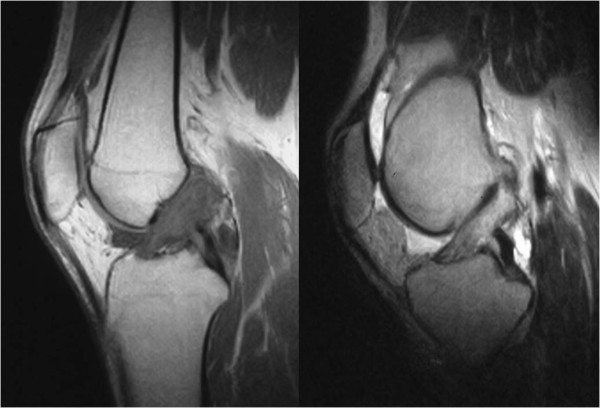
**Preoperative MRI image**. ACL presented signal changes of iso- and high signal intensity at T1WI (left) and at high signal intensity at T2WI (right), and also demonstrated swelling.

No signs were detected in radiographic findings at the initial visit and no calcifications were found within the joint (Figure 1). Magnetic resonance imaging (MRI) showed the ACL was swollen and iso-signals to high-signals at TIWI and high-signals at T2WI, indicating significant signal changes compared to the MRI of a normal ACL. No findings that suggested meniscus damage and cartilage damage were found, and no abnormal findings in the synovium at other sites were detected by MRI (Figure 2).

Although some lesions in the ACL were clearly detected, no definite diagnosis was made, so arthroscopy was performed in the winter of 2004 for diagnosis and treatment.

**Figure 3:**
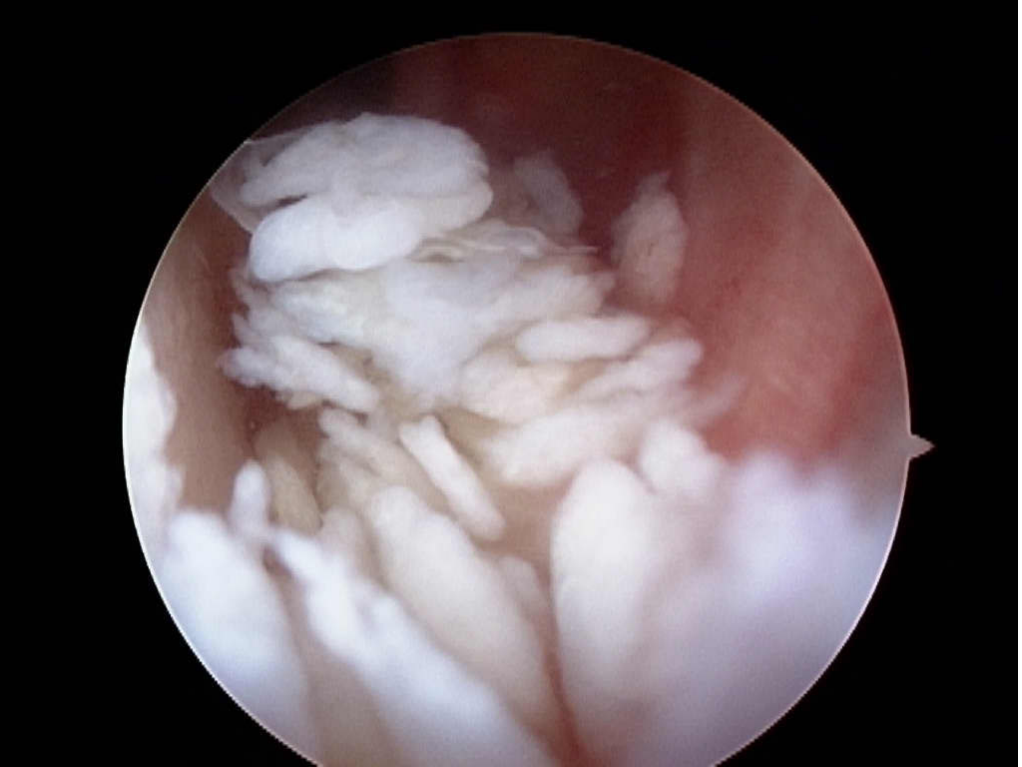
**Arthroscopic finding of lateral gutter**. Numerous rice grain-sized tissues, which seemed to be cartilaginous segments, were present in the joint.

**Figure 4:**
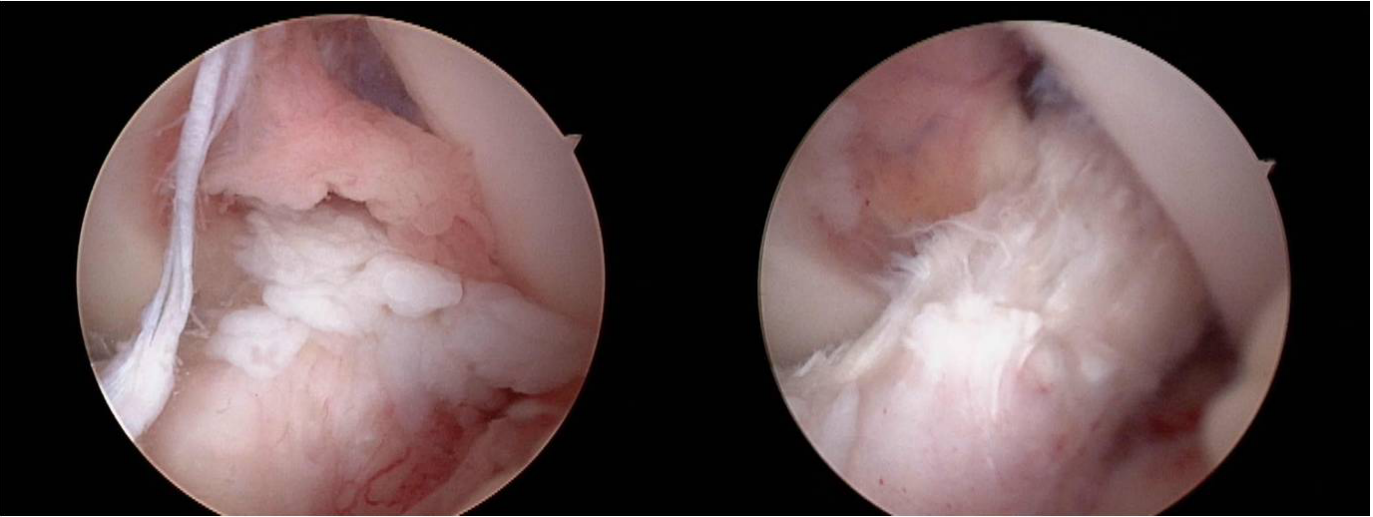
**a) Arthroscopic finding of intercondylar cavity**. Cartilaginous segments were present in the synovium on the ACL surface. b) ACL after arthroscopic synovialectomy.

Numerous white rice-grain size free bodies, presenting the so-called "snow storm appearance" [5] were observed inside the articular cavity of the left knee (Figure 3). Proliferation of the synovium on the ACL was identified and cartilaginous tissue was included (Figure 4a). Although there were numerous free bodies, no proliferation was found in the synovium except around the ACL, and no damage was found in the meniscus and cartilage. The synovium that proliferated around the ACL was removed as much as possible using a shaver and forceps, and free bodies that were assumed to be cartilaginous segments were washed out (Figure 4b).

**Figure 5:**
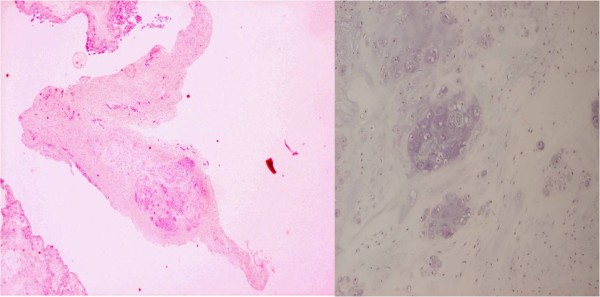
**Pathohistological finding**. a) × 20. Hematoxylin Eosin staining. A cartilaginous tissue enclosed by the synovial tissue was detected. b) × 300. Hematoxylin Eosin staining.

A small nodular-shaped tissue enclosed by a thin synovium was detected on pathohistological images (Figure 5). No signs of ossification were found, and the pathological diagnosis was synovial chondromatosis.

**Figure 6:**
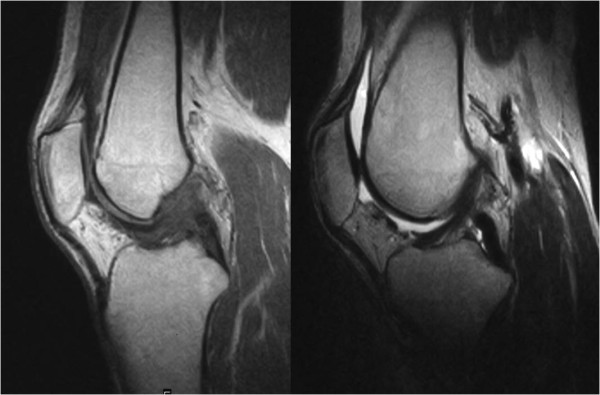
**Postoperative MRI (T1WI (left) and T2WI (right))**. ACL presented normal signal intensity.

Pain in the left knee rapidly decreased after the operation and full weight bearing was started at postoperative day one. According to MRI taken at postoperative 9^th ^month, the ACL presented normal signal intensity and no thickening of other synovia was found (Figure 6). Four years after surgery, there was no recurrence of pain in the left-knee. The left-knee ROM indicated an extension of 0° and a flexion of 145°, which indicated regaining of normal ROM and no recurrence of locking-like symptoms. The results of Lachman test and anterior drawer test were negative.

## Discussion

With respect to cases of synovial chondromatosis originating from the cruciate ligament in the knee, only 2 cases originating from the posterior cruciate ligament have been reported [3,4] and no case has been reported of occurrence originating from the anterior cruciate ligament. Synovial chondromatosis is considered to be a disease wherein mesenchymal cells under the overlying cells of the synovial tissue of joints, peritendons, and synovial capsule cause metaplasia to be changed to cartilaginous tissue. According to the stage classification of synovial chondromatosis reported by Miligram [6], phase 1 is the stage with synovial lesions, phase 2 is the stage with a combination of synovial lesions and free bodies, and phase 3 involves the presence of numerous free bodies.

The theory that the etiology of synovial chondromatosis originates in synovium is the major thrust. However, Coolican *et al*. [7] and Kay *et al*. [5] suggested that the etiology of chondromatosis is present in free cartilage and it was hypothesized that a cause of this disease is the proliferation of chondrocytes within the articular fluid with free bodies subsequently incorporated into the synovium. In the present case, significant proliferation of the synovium was found only around the ACL. In the pathological findings of the present case, cartilaginous segments were present in the synovium of the ACL. Further, no synovial proliferation was found other than around the synovium of ACL. We did not find any significant chondral lesions at arthroscopy. Therefore, it is reasonable to think that in the present case synovial chondromatosis originated from synovium around the ACL.

Preoperative diagnosis was difficult for this case. Although various diseases causing locking symptoms such as meniscus damage, osteoarthritis, osteochondritis dissecans, osteochondral fractures, discoid meniscus, synovial cysts, pigmented villonodular synovitis were considered for identification, the clinical symptoms did not completely agree with any of them. It was clear from MRI that there were some lesions in the ACL because of abnormal signals in the ACL. However, no report was available on synovial chondromatosis originating from the ACL and it was difficult to make a preoperative diagnosis of synovial chondromatosis at this point. In radiological viewpoint, correct diagnosis was difficult preoperatively, because synovial chondromatosis may have nonspecific imaging findings on MRI as seen in this case. Intraarticular free bodies detected arthroscopically were not clearly visualized on MRI, as they had similar signal intensity to joint fluid. Previously reported case of synovial chondromatosis arising from PCL also had nonspecific imaging findings of intraarticular lesion surrounding PCL without alteration of PCL morphology and signal [4]. In this case, swelling of ACL with poor visualization of ACL fibers accompanied by adjacent cystic lesions on T2-weighted image are reminiscent of ganglion cyst with coincident mucoid degeneration of ACL [8,9]. Complete or incomplete tear of ACL may have similar imaging findings [9]. Arthroscopy was considered most appropriate for a definite diagnosis of the present case.

In the treatment of synovial chondromatosis it has been reported that arthroscopic discharge of free bodies is considered to be sufficient so synovectomy is not necessary due to problems with arthrotomy in functionality such as postoperative restriction of ROM [1,10]. On the other hand, there was a report suggesting combination with synovectomy [11]. In the present case when numerous cartilaginous segments are present in the synovium, it is technically easier to remove cartilaginous segments along with the synovium. As long as the synovium is considered to be a cause of the expression of the present disease, it seems desirable to remove as much synovium as possible.

Because the observation period after treatment of this case was short, we must observe the outcome very carefully with respect to recurrence of the disease in the future. Because the synovium around the ACL was dissected, the blood flow was temporarily reduced so there is a possibility that loosening of the ACL may increase during the process of remodeling. It is important to continue long-term observation of the outcome regarding the functions of the ACL.

## Conclusion

A case of synovial chondromatosis originating from the synovium of the ACL that resulted in a mechanical block to knee extension is reported. Arthroscopy is useful to obtain a definite diagnosis and treatment. Physicians should keep in mind the present pathology when making a differential diagnosis of a patient with locking of the knee.

## Consent

Written informed consent was obtained from the patient for publication of this case report and any accompanying images. A copy of the written consent is available for review from the Editor-in-Chief of this journal.

## Competing interests

Each author certifies that he has no commercial associations (e.g. consultations, stock ownership, equity interest, patent/licensing arrangements, etc) that might pose a conflict of interest in connection with the submitted article.

## Authors' contributions

TM carried out the treatment and followed up the patient. TK participated in the radiological assessment and advised the differential diagnosis in a view point of MRI. KS picked up the patient at outpatient clinic, and participated in the present report coordination. All authors read and approved the final manuscript.
